# Better Understanding of the Metamorphosis of Pregnancy (BUMP): protocol for a digital feasibility study in women from preconception to postpartum

**DOI:** 10.1038/s41746-022-00579-9

**Published:** 2022-03-30

**Authors:** S. M. Goodday, E. Karlin, A. Brooks, C. Chapman, D. R. Karlin, L. Foschini, E. Kipping, M. Wildman, M. Francis, H. Greenman, Li Li, E. Schadt, M. Ghassemi, A. Goldenberg, F. Cormack, N. Taptiklis, C. Centen, S. Smith, S. Friend

**Affiliations:** 1 4YouandMe, Seattle, WA USA; 2 Department of Psychiatry, University of Oxford, Oxford, UK; 3 MindMed, Inc., New York, NY USA; 4 Tufts University School of Medicine, Boston, MA USA; 5 Evidation Health Inc., Santa Mateo, CA USA; 6 Sema4, Stamford, CT USA; 7 Institute for Medical Engineering and Science, MIT, Cambridge, MA USA; 8 Department of Electrical Engineering and Computer Science, MIT, Cambridge, MA USA; 9 Vector Institute, CIFAR AI Chair, Toronto, Canada; 10 SickKids Research Institute, Department of Computer Science, University of Toronto, Toronto, Canada; 11 Cambridge Cognition, Cambridge, GB USA; 12 Department of Psychiatry, University of Cambridge, Cambridge, GB USA; 13 Bodyport Inc., San Francisco, CA USA

**Keywords:** Reproductive signs and symptoms, Reproductive biology

## Abstract

The Better Understanding the Metamorphosis of Pregnancy (BUMP) study is a longitudinal feasibility study aimed to gain a deeper understanding of the pre-pregnancy and pregnancy symptom experience using digital tools. The present paper describes the protocol for the BUMP study. Over 1000 participants are being recruited through a patient provider-platform and through other channels in the United States (US). Participants in a preconception cohort (BUMP-C) are followed for 6 months, or until conception, while participants in a pregnancy cohort (BUMP) are followed into their fourth trimester. Participants are provided with a smart ring, a smartwatch (BUMP only), and a smart scale (BUMP only) alongside cohort-specific study apps. Participant centric engagement strategies are used that aim to co-design the digital approach with participants while providing knowledge and support. The BUMP study is intended to lay the foundational work for a larger study to determine whether participant co-designed digital tools can be used to detect, track and return multimodal symptoms during the perinatal window to inform individual level symptom trajectories.

## Introduction

Despite major advancements in prenatal care over the past several decades, maternal health remains a significant global public health problem associated with considerable morbidity, mortality, and economic burden^1,2^. The incidence of maternal disorders approached 80 million cases in 2017, globally^2,3^, while the United States (US) has the highest maternal mortality compared to other developed countries^4^. Women are attempting to get pregnant at later ages when risks of pregnancy-related complications and conception problems are higher^5^. To mitigate the risks of pregnancy, pregnant women are monitored frequently through periodic in-clinic visits with their clinician. However, little is known about the progression of symptoms between clinic visits. While there are symptoms that are specific to being pregnant because of complex hormonal variations such as nausea, fatigue, or shortness of breath, the symptoms experienced are broad and highly varied in frequency and severity. There is a striking lack of knowledge into the pathobiology of pregnancy-related severe symptoms, complications, and diseases and why some women experience worse trajectories than others^6,7^. This knowledge deficit is in part due to the lack of methodologies to adequately measure both the scope and needed resolution of symptoms in the complex perinatal environment. Accordingly, most research has focused on aggregated data and not objective individual-level symptoms that could define individual health journeys. Pregnancy symptoms, complications, and diseases result from entangled interactions between past susceptibilities and vulnerabilities, (e.g., each woman has a distinctive baseline risk with differing organ and mental fragility), and current exposures (e.g., stress, diet, activity, environment, psychosocial support)^7–11^. This complex etiology makes each experience unique and challenging to inform when focusing on aggregate, average experiences across women.

**Fig. 1:**
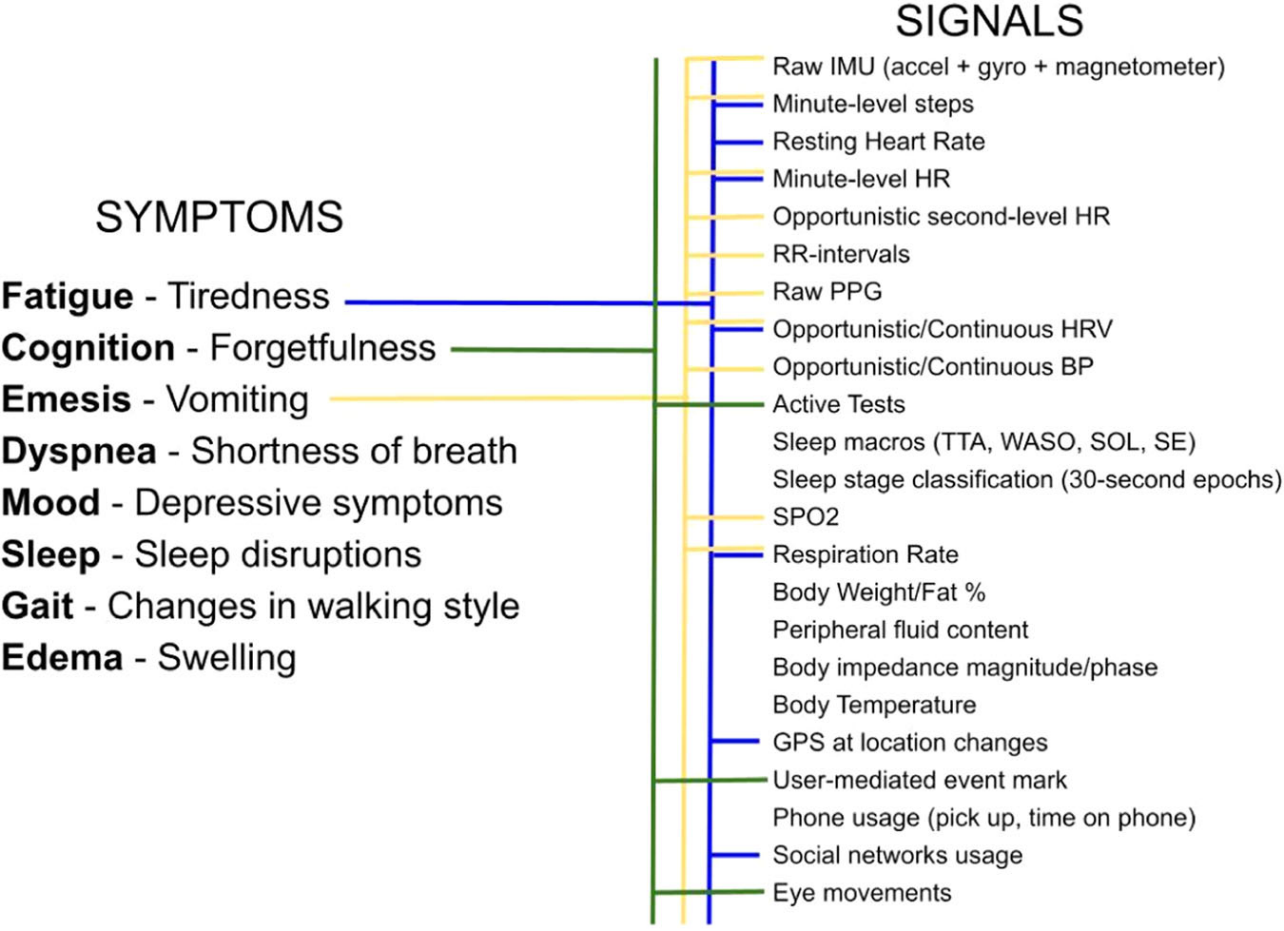
Pregnancy-related symptoms to objectively measured signals. A spider chart of pregnancy-related symptoms and their potential objective signals from digital devices.

Semi-ubiquitous digital devices offer possible upstream prevention tools to track individual-level objective symptoms during the perinatal window and uniquely return this information to the user. Consumer-grade wearable devices such as smartwatches and smart rings are capable of tracking semi-continuous measures of physiology (heart rate, heart rate variability, body temperature, blood pressure, and oxygen saturation) and behavior (activity, relative location, and sleep duration and quality)^12^. The tracking of such physiologic metrics has obvious advantages for the potential early detection of pregnancy-related conditions such as preeclampsia and gestational hypertension^13,14^. The most common application of digital technology during pregnancy is activity and heart rate tracking (e.g., Grym et al.^15^), yet the combined use of different tools could achieve much more. Collectively, these devices could detect objective measures of broad pregnancy symptoms such as those routinely captured during aperiodic clinic visits (e.g., blood pressure or heart rate) but at much higher resolutions in addition to several other symptoms that lack adequate clinical assessments such as cognition, mood, and sleep. It is the combination of tracking broad, multimodal symptoms (Fig. 1) at the individual level and returning this information to women in near-real time, while providing high-resolution information for healthcare professionals that highlights the promise of these tools for personalized health tracking and with the pairing of artificial intelligence, possibly early intervention, and prevention of pregnancy-related health problems.

The goal of the Better Understanding the Metamorphosis of Pregnancy (BUMP) study is to gain a deeper understanding of the pre-pregnancy and pregnancy individual-level symptom experience through the use of wearable devices, smart scales and smartphone apps. This paper presents the protocol for the BUMP study.

## Methods

### Study design

The BUMP study is a digital health longitudinal feasibility study conducted by 4YouandMe (www.4youandme.org), a non-profit that conducts open source, digital health research. The BUMP study was designed to follow a target sample size of 1000 women from preconception to the fourth trimester. Multiple digital tools including a smartwatch, smart ring, smart scale, and study smartphone apps will be used to detect and track self-reported subjective and objectively measured symptoms associated with hormonal variations in women who are trying to conceive, pregnant, and in the postpartum period. This study was approved by the IRB, Advarra (Pro00047893).

The specific objectives of the BUMP study are to:

1. Demonstrate the feasibility of the collection and integration of digital and clinical data from pregnant women or women trying to conceive using engagement strategies focused on support and knowledge
2. Characterize the inter/intra individual variation in objective and subjective pregnancy-related symptoms and signs (blood pressure changes, emesis, fatigue, mood, sleep, dyspnea, cognition, edema, gait, and other)
3. Characterize the inter-/intra-individual variation in objective and subjective pre-pregnancy-related symptoms and signs associated with fertility treatments (i.e., mood, cognition, sleep, anxiety, irritability, anger, fatigue, vasomotor, other)
4. Determine individual-level patterns in subjective and objective data that are associated with pregnancy-related complications, severe symptoms, and diseases

### Recruitment

Enrollment began on 23 February 2021, and will continue until December 2022. Participants are recruited over Sema4’s patient portal (WIRB Protocol No. - Sema4-WIRB-002). Sema4’s patient portal provides a mechanism for Sema4 to engage with its patients, including communicating Sema4’s test results and managing electronic consents. This platform also comprises a triggered digital consent platform that requests patient users’ permission to access electronic medical record (EMR) data, so that Sema4 can return such data through the Sema4 patient portal in a patient-centric format with insights specific to the patient’s health journey; and for research purposes, such as to be re-contacted to participate in specific and relevant research-related programs. Patients who completed a Sema4 test commonly associated with pregnancy (e.g., non-invasive prenatal testing, expanded carrier screening) and consented to have their EMR data accessed by Sema4 receive an email notification from Sema4 with a brief description of the study and option to provide contact information so that a study representative can contact them. We conducted A/B testing of different recruitment emails to determine which language had the most success in inciting interest from potential participants. A sample of one of these recruitment emails can be found in the supplementary information (Supplementary Note 1). Participants may also be recruited from other channels, including social media, clinics and word of mouth with a particular emphasis on targeting women from more diverse and disadvantaged backgrounds. The same inclusion criteria and consent process will occur for these additional participants with the exception of EMR data access. For these cases, EMR data will be accessed using a 3rd party company, HumanAPI that securely retrieves participants’ EMR data from their healthcare providers, negating the need for consent to have Sema4 access and retrieve this information.

The BUMP study comprises two cohorts of women. The BUMP cohort includes women who are currently pregnant, and the BUMP-Conception (C) cohort includes women actively attempting to get pregnant. There is very little data on the early stages of pregnancy, largely a result of recruitment challenges. Many women are unaware of very early pregnancy, while miscarriage rates are highest during the early pregnancy phase. Recruiting a preconception cohort enables the collection of information during the first few weeks of pregnancy which could lead to informing determinants of miscarriage, in addition to providing data for a baseline prior to pregnancy to better inform pregnancy-related symptom trajectories.

BUMP participants are enrolled if they are pregnant and asked to participate into their fourth trimester (three months postpartum). To be included in the BUMP cohort participants must be up to and including 15 weeks pregnant, be 18+ years of age, own a personal smartphone that is an iPhone SE or newer or an Android version 6.0 device or newer, have an unshared email address and be proficient in English. Participants are excluded if they do not have a permanent address, are unable to read or understand the study materials, or intend to terminate their pregnancy at time of enrollment. Eligible and consented participants are mailed an Oura smart ring (https://ouraring.com/) and a Garmin Venue Sq smart watch (https://www.garmin.com/) and asked to continuously wear these devices for the duration of their participation alongside interacting with a BUMP study app. Participants are also provided with a Bodyport cardiac scale (https://bodyport.com/) for daily weigh-ins.

BUMP-C participants are enrolled if they are actively trying to conceive and will be followed for six months or until conception, whichever comes first. These participants have the option of transitioning to the BUMP cohort if they become pregnant. Trying to conceive is defined by a variety of methods including spontaneous conception, or any form of fertility treatment that could include oral/injectable treatments or other assisted reproductive treatments such as In vitro fertilization (IVF). The same inclusion/exclusion criteria applied to BUMP apply to BUMP-C except for including women currently actively trying to conceive and those 18–40 years of age. BUMP-C participants are mailed an Oura smart ring and asked to wear this continuously for the duration of their participation alongside interacting with a BUMP-C study app.

### Study smartphone app

**Fig. 2:**
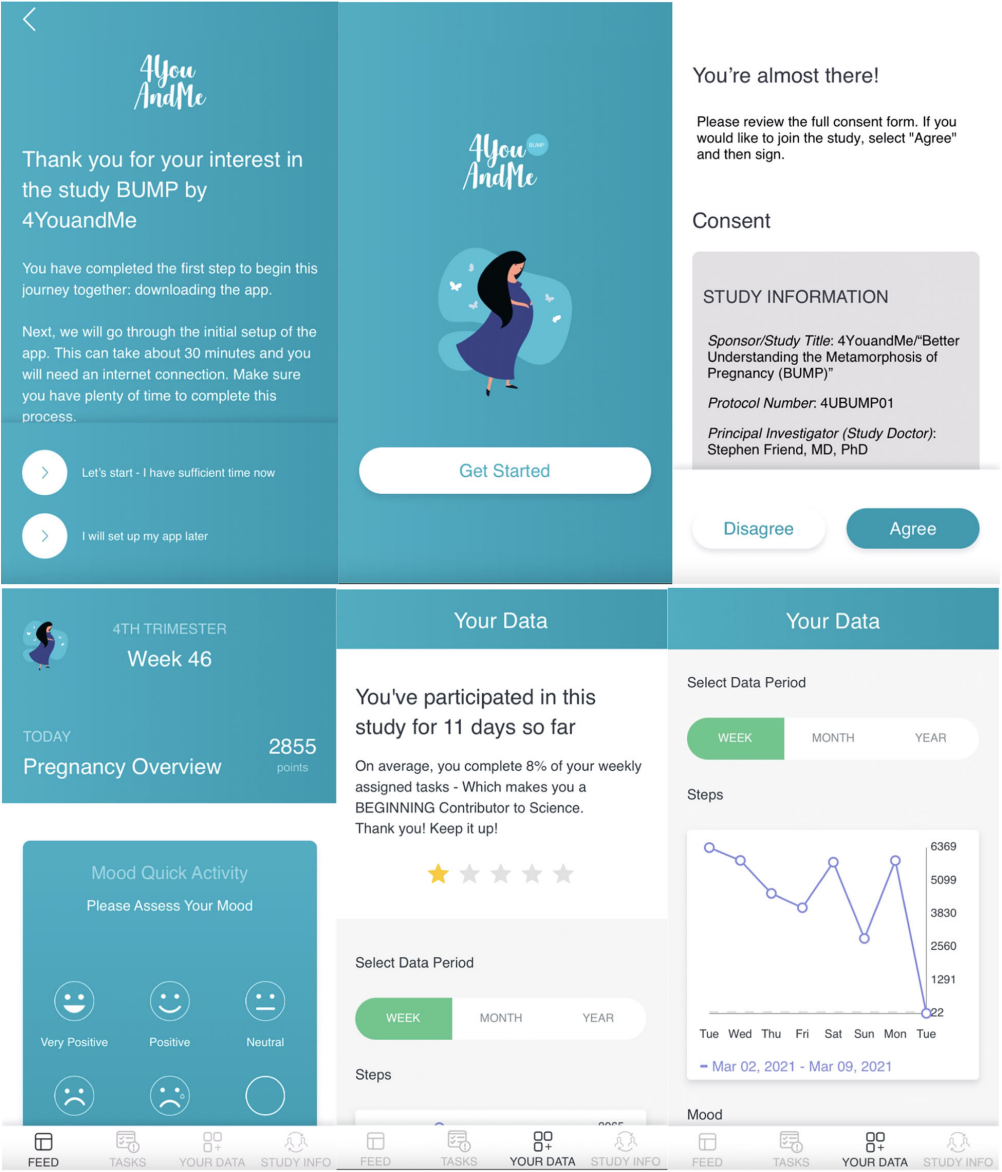
Sample screenshots from the BUMP study app. Select sample screenshots from the BUMP study app onboarding and e-consent (top 3 panels), quick activities (ecological momentary assessments) (bottom left panel), and the ‘Your Data’ tab that tracks participation and returns select symptom summaries back to participants (bottom middle and right panel).

Two study smartphone apps (BUMP and BUMP-C) were developed to serve as participants’ hub for all study-related communications and data collection (Fig. 2). The app is equipped with daily and intermittent surveys and active tasks measuring key symptoms associated with hormonal variations in women attempting to get pregnant, or currently pregnant. In pregnant women we targeted nine key symptoms of pregnancy including: changes in mood, sleep, cognition, blood pressure (BP), gait, nausea/vomiting (emesis), shortness of breath (dyspnea), swelling (edema), and fatigue. In women attempting to conceive, we targeted similar symptoms (a complete list of app-based survey and active tasks content can be found in Table 1) that might arise from fertility treatments such as vasomotor symptoms. Participants are instructed to download the app from the App Store (iOS) or Play Store (Android). Upon download, participants are walked through the study onboarding process, starting with eConsent. ResearchKit’s eConsent framework (http://researchkit.org/docs/docs/InformedConsent/InformedConsent.html) was used (for iOS) that includes brief summary screens with a link out to a ‘learn more’ section to convey the consent narrative in a more digestible way. Participants are provided with the full eConsent to review, followed by a brief comprehension quiz before digitally signing and are immediately sent a signed version via their provided email address. Participants are also walked through downloading and agreeing to the manufacturing terms of the included third party applications (described below). A 4YouandMe engagement specialist assists participants with this process over the phone and describes what to expect.

**Table 1:** Active study app surveys and tasks.

Measure	Cadence	Assessments	Source	Cohort
Active (survey)	Baseline (+35 days)	Demographics, lifestyle, and medical history^a^	Study app	Both
		Medications^a^	ES check in	Both
		Adverse Childhood Events (ACE)^31^	ES check in	BUMP
		Perinatal post-traumatic stress disorder^32^	ES check in	BUMP
		Ten-item personality inventory^33^	Study app	Both
	Daily	Daily Stress and Symptom Survey^a^	Study app	Both
	Daily	EMA (mood, cognition, stress, energy [anger, irritability, anxiety BUMP-C only])^a^	Study app	Both
	Monthly/weekly	Healthcare/fertility treatment utilization^a^	Study app	Both
	Weekly	Pitting Edema Assessment^a^	ES check in	BUMP
	Weekly	Pregnancy-Unique Quantification of Emesis^34^	Study app	BUMP
	Weekly	PROMIS Sleep-Related Impairment (V1.08a)^35^	Study app	Both
	Weekly	Fatigue Assessment Scale^36^	Study app	Both
	Every 2 weeks	Patient Health Questionnaire-9^37^	ES check in	Both
	Triggered	Columbia-Suicide Severity Rating Scale^38^	ES check in	Both
	Every 2 weeks	General Anxiety Disorder-7^39^	ES check in	Both
	Monthly	PROMIS Global-10^40^	Study app	BUMP
	Monthly	PROMIS Emotional Support (4a)^41^	Study app	BUMP
	Post-natal	Maternal Social Support Index^42^	ES check in	BUMP
	Post-natal	Pediatric Outcomes^43^ and birthing data^a^	ES check in	BUMP
	Post-natal	Edinburgh Postnatal Depression Scale^44^	ES check in	BUMP
Active (Tasks)	Every other day	Cogkit N-Back Test^17^	Cambridge cognition	BUMP
	Every 4 days	CANTAB Psychomotor Vigilance Test (PVT; https://www.cambridgecognition.com)		BUMP
	Weekly	CANTAB Emotional Bias Test (EBT)^16^		Both
	Weekly	Reaction Time Test/Trail Making Task (http://researchkit.org/docs/docs/ActiveTasks/ActiveTasks.html)	Study app	BUMP-C
	Every 2 weeks/monthly	2-Minute Walk Test (http://researchkit.org/docs/docs/ActiveTasks/ActiveTasks.html) and Gati Task(58)	Study app	BUMP
	Every 2 weeks/weekly	Video diary	Study app	Both

*PROMIS* Patient-Reported Outcomes Measurement Information System, *EMA* ecological momentary assessment, *ES* Engagement Specialist.

^a^4YouandMe developed questionnaire.

The study app (Fig. 2) was designed to engage participants in daily survey and active task completion with push notifications and daily rewards that are tallied at the end of the study in the form of modest financial compensation. Surveys included self-reported momentary, daily and intermittent measures of symptoms. Active tasks include cognitive and emotional bias tasks (Cambridge Cognition; https://www.cambridgecognition.com), video diaries for the collection of voice and face data (Table 1), a gait task to measure changes in gait throughout the data collection period, and a walk test for measuring cardiovascular fitness and recovery. Active tasks and surveys are uniquely scheduled on different days to balance the day-to-day participation so that the average daily burden does not exceed five minutes. Once participants start using the app, they are presented with a dashboard that enables them to track select subjective and objective symptoms over time (Fig. 2).

Some survey content is sensitive and includes questions about suicide-related thoughts. These sensitive measures (Table 1) are administered over the phone by 4YyouandMe engagement specialists. In the event of a suicide risk event, an on-call study psychiatrist is triggered immediately to call the participant to facilitate the necessary care.

### Cognitive Active tasks

CANTAB EBT^16^ detects perceptual bias in facial emotion perception. Fifteen computer-morphed images derived from the facial features of real individuals that are morphed between two emotions (e.g., happy and sad) of varied intensities are presented to the participant one at a time on-screen. Each face is displayed for 150 ms and presented three times. The participant must then indicate which emotion they think the face displayed from two options (happy and sad), presented on-screen.

The Cognition Kit N-Back^17^ is a measure of attention and working memory. In each testing session nine symbols, randomly selected from a pool of 227, are presented for 600 ms, one at a time for 30 trials. Participants were asked to respond when any symbol is the same as the symbol presented two trials previously.

The Psychomotor Vigilance Task (PVT; https://www.cambridgecognition.com) assesses vigilance and reaction time. Participants must monitor the smartphone screen until a number appears. The participant should touch the screen as soon as they see the numbers appear. The number in the box at the center of the screen increases each msec, until the participant touches the screen, providing immediate feedback on reaction time. 45 trials are presented with a variable inter-trial interval.

### Wearable devices

Participants are asked during in-app onboarding to perform a one-time syncing of their provided device to the associated smartphone app. This task can be performed at a later time as well, while participants wait for their devices to be delivered in the mail. BUMP-C participants are given a choice to keep their Oura ring, or to receive financial compensation proportionate to their accumulated study points for task completion. BUMP participants receive financial compensation proportionate to their accumulated study points for task completion and can keep their Oura smart ring or Garmin smartwatch. If participants withdraw from the study early, they will be asked to return their device(s).

The Oura smart ring 2 (https://ouraring.com/) is a light-weight device that is worn on the finger and collects the following passive data: nighttime relative body temperature, sleep quantity and quality, resting heart rate (RHR), heart rate variability (HRV), respiration rate (RR), and activity (steps).

The Garmin Venu Sq (https://www.garmin.com/) smartwatch is made of a flexible, durable material similar to that used in many sports watches and includes a stainless steel watch case and buckle. The Garmin smartwatch collects the following passive data: RHR, HRV, RR, Pulse Ox blood oxygen saturation monitor and sleep quantity.

The Bodyport cardiac scale (https://bodyport.com/) comprises a physical platform on which the user stands with bare feet. Four electrodes located on the top surface of the platform are used to obtain three biological signals from the user’s body. The first is a passive electrical signal and is similar in origin to an electrocardiograph. The second reflects pulsatile blood flow and is determined by measuring small changes in the electrical resistance through the legs. This signal is obtained by transmitting a small, safe, battery-generated current between the user’s feet. The third signal measures blood flow velocity through the Aorta and reflects mechanical function of the heart. From these signals, the Bodyport cardiac scale measures multiple biomarkers including weight, peripheral fluid levels, HR, HRV, cardiac time intervals, and ejection forces.

### Third party applications and additional passive assessments

During the onboarding, participants are asked to download the apps corresponding to each study device. Additionally, participants in BUMP are given the option to use RescueTime – an application that tracks screen time and are asked if they would like to grant permission to the study investigators to access their Twitter and Instagram data (e.g., numbers of posts, like, follows, followers, tags). Participants are also asked if investigators can access their relative location data.

### Digital engagement strategy

Engagement is typically poor in digital health app-based studies^18–20^, and is particularly challenging when studies lack a clinical referral for recruitment. 4YouandMe has been piloting digital engagement strategies across other studies using participant centric principles that aim to provide knowledge and support to research participants through a variety of channels as described below.

Participants are paired with an ‘Engagement Specialist’ at the beginning of the study and are asked to engage in phone or video calls approximately every two weeks to every month. These calls serve multiple purposes: (1) to offer support to participants and troubleshoot digital device and study-related problems; (2) to collect qualitative data on the pre-pregnancy and pregnancy experience with a particular emphasis on collecting contextual data on why women perceive shifts in symptoms, or notice shifts in objective symptoms from their returned symptom data; and (3) to solicit feedback and suggestions from participants as part of the co-design of the app and the approach. At this feasibility stage, we employ a ‘learning by doing’ approach whereby direct participant feedback is used to refine the app and overall study experience in real time during the course of active study time.

The study investigators will hold study-related events that include joint investigator/participant anonymous zoom calls that aim to give participants an update on study progress, share some preliminary results and give the participants a chance to meet the study team, ask questions and offer further feedback on study experience.

### Analysis plan

Individual symptom maps will be curated for each participant including multimodal subjective and objective data streams across digital devices. We plan to use a variety of supervised and unsupervised machine learning techniques to identify and validate multimodal digital biomarkers and create predictive models for future participant state in all participants, and use model ablation to identify signals with high predictive value (e.g. refs. ^21–23^). Data integration^24,25^ will involve statistical exploratory analyses on self-reported subjective vs. objective measures of pregnancy symptoms and will integrate different types of data into similarity networks (e.g., combining different measures of pregnancy symptoms such as from text and image (face) data). We will explore Embedding/Latent State Discovery using neural networks and autoregressive methods used to assess if there is convergence of particular patterns in combined objectively measured sensor data across observed individuals^26,27^. State Transition Detection will be used to identify pregnant women transitioning from symptoms to more severe symptoms and complications

### Data management

All participant data will be securely stored in coded form, stripped of personal identifiers using a unique identifier. Personally identifiable information will be stored in a separate location to coded study data and destroyed ten years after study completion. Personally identifiable information will be kept strictly confidential and only accessible to authorized members of the immediate research team. All data collected during the study will be performed by Evidation Health (https://evidation.com/), a health and measurement company in accordance with local and state laws, regulations, and IRB policies around study data collection and use. Data will be transmitted using Transport Layer Security (TLS) and stored on encrypted disks on secure servers. Administrative access to these servers is limited to only the necessary IT staff at Evidation Health. The 4YouandMe server at Evidation collects data from the participant’s wearable sensors, like Oura. Authentication and authorization is managed through OAUTH2 over HTTPS from the respective vendor’s website. Data is retrieved directly from vendor servers through their public APIs and sent to Evidation, where all processing is conducted.

Data recorded during Engagement Specialist check-in calls is collected and managed in a REDCap database. Research Electronic Data Capture (REDCap^28,29^) is an electronic data capture tool hosted and managed by the Center for International Emergency Medical Services (CIEMS; http://ciems.org/). REDCap is a secure, web-based software platform designed to support data capture for research studies. The REDCap database can only be accessed by authorized users from the 4YouandMe research team. Coded (stripped of personally identifiable information) EMR data will be provided to Evidation Health by Sema4 using a study-specific participant ID to link EMR data to study app data on the Evidation platform. EMR data may also be accessed through other channels. Active tasks, survey and device data will be collected and managed through the Evidation Health data platform. The study data files are accessible to authorized research personnel who work in relevant components of the study.

## Discussion and expected impact

Women transitioning to a pregnancy state could be better supported and empowered. Sadly, similar to other female reproductive transitions such as menopause, pregnancy is still an enigmatic experience. Many pregnancy-related complications could be prevented if detected earlier. Maternal health has widespread impact not only for the mother’s immediate health and well-being but also for long-term postnatal, fetal and infant health^6^. Targeting the perinatal window for optimal maternal health is arguably one of the most upstream preventive approaches to setting a healthy playing field for children’s lifelong development.

The application of digital technology for healthier pregnancy transitions has started to emerge, including activity trackers to encourage physical activity and to track diet and other physiological measures such as heart rate, heart rate variability, body temperature and blood pressure (https://www.whoop.com/thelocker/wvu-study-pregnancy-health-trends/; https://www.purdue.edu/newsroom/releases/2018/Q3/wearable-technology-could-help-pregnant-women-detect-health-complications,-improve-outcomes.html)^15^. Yet the feasibility and effectiveness of these tools in achieving healthier pregnancies remains to be seen^30^, particularly across diverse and disadvantaged populations. Numerous smartphone apps exist that provide knowledge to women about what to expect during pregnancy. However, we still have limited information on the broad spectrum of pregnancy experiences, largely because we have not had adequate tools to track this complex experience. Much of the knowledge on “what to expect” comes from aggregated data of average experiences in women. While this information might be useful for some women, it may not reflect the experience of all women.

The BUMP study is intended to serve as a participant-centric feasibility study precursor to a larger main study. The uniqueness of the BUMP study is in the use of: (1) a multimodal approach with a focus on individual-level symptom trajectories; (2) the participant centric, co-design strategy; (3) a data capture window from preconception to the postpartum period; and (4) an enriched data set with high-frequency self-reported measures alongside clinically collected data.

A multimodal approach (Fig. 1) aims to leverage machine learning and artificial intelligence assessments to integrate and determine the impact of various features on the individual level risk of a particular outcome (symptom, or state). This approach has advantages for deriving new insights into poorly understood and complex disease pathways and is well suited to the complexity of individual-level pregnancy experience. Women enter the perinatal window with different baselines resulting from complex interactions between genetics and past experiences, while their symptom experiences are modified by psychosocial factors during this period^7–11^. Integrated digital tools have the potential to provide individual-level information to women, relative to their baselines and their specific context. These approaches could inform women when there is a deviation from their ‘normal’. Given the wide range of behavioral and physiological features captured, this information could be used for both physical and mental health insights during the perinatal window (i.e., in the longitudinal tracking of mood). Eventually, with the use of machine learning clustering approaches and large enough datasets, we might be able to identify archetypes (sub-groups) of women who follow similar patterns of symptoms where new and more accurate treatments could be applied to these groups. This individual trajectory approach could tackle the shortcomings of the aggregate data approach that makes erroneous assumptions about generalizability, often producing findings that are non-translatable to under-represented groups in research, and thereby contributing to socioeconomic and racial maternal health disparities. Although the study population recruited into the BUMP feasibility study is likely to not be fully generalizable to diverse populations, this study does involve targeted recruitment material that will attempt to recruit women via social media and other outlets from more diverse backgrounds. The purpose of this initial study is to work through challenges in the implementation of the proposed digital approach. The main BUMP study will involve a targeted recruitment strategy and partnerships with community-based organizations to ensure a diverse study sample that is crucially needed for perinatal and digital health research.

While the excitement for the utility of digital technology for healthier pregnancies is clearly present, a major unresolved challenge is engagement, as seen in other areas of digital health^18–20^, Given these tools will ultimately be used by individuals and not their healthcare providers, extensive work is needed to design tools that are patient and women centric, while providing the necessary support. The BUMP study employs a patient-centric co-design strategy, where women are invited to help shape the app, the approach and ultimately their study experience. This co-designed approach is intended to both inform the methodology in the main study, and potentially help shape the tools that might result from this, and others work.

In following women from preconception to the postpartum period, we are able to capture a baseline A state, a pregnancy B state, and a postpartum C state. Some symptoms will dramatically subside during the postpartum period (e.g., shortness of breath), while others may increase (e.g., mood). The transitions through these states enable rich data capture for testing the feasibility of picking up objectively measured signals from sensor data.

Digital health studies that collect objectively measured data require ground truth measures to validate the accuracy of the sensor data. The BUMP study involves the collection of high-frequency self-reported symptom data, qualitative data of symptom context collected during engagement specialist check-ins, and EMR clinical data providing an enriched data set to validate the accuracy of the objectively measured data captured from the study devices.

The BUMP study’s long-term goals and potential implications are in the development of a digital personalized health tracker that provides both knowledge and support to women entering the metamorphosis of pregnancy. The prospect of returning objectively measured information to women outside the medical system has the potential for empowering tools that could help women predict when symptoms might become risky and be a sign of an impending complication or condition within their individualized context. This empowerment, enabled by monitoring of symptoms and AI driven insights provides opportunities for personalized lifestyle interventions as opposed to relying on pharma-driven acute treatments that could have a dramatic impact globally on pregnancy-related outcomes. Additionally, this approach may allow women, particularly minority women, to better advocate for themselves in a system where they are subject to the implicit biases of healthcare systems and providers. The intention is to continue to co-design this work with women to facilitate translation of findings to all women, particularly those who may have less access to digital health tools and equitable health care.

### Reporting summary

Further information on research design is available in the Nature Research Reporting Summary linked to this article.

## Supplementary information

Supplementary Information

Reporting Summary

## Data Availability

It is 4YouandMe’s mission to host all generated study data into the public domain so that any ‘qualified’ researcher can access it. Accordingly, among participants who opt-in, coded study data from the BUMP study participants will be placed onto the Synapse platform (synapse.org) at Sage Bionetworks (https://sagebionetworks.org) and can be freely accessed by any researcher who becomes ‘qualified’ by becoming a registered and certified Synapse user (https://help.synapse.org/docs/User-Account-Tiers.2007072795.html), and by meeting the specific conditions of use that require submitting an intended data use statement alongside an IRB approved protocol. This coded data set will not include sensitive data including relative location, video diaries, social media data, and qualitative data captured during Engagement Specialist check-in calls. The BUMP study is still active (as of the date of this publication). Once the study is complete, data will be hosted on the Synapse platform. Please check the 4YouandMe website (www.4youandme.org) or contact the corresponding author (sarah@4youandme.org) for an update on when study data will be available and how to access.

## Code availability

After the BUMP study is complete any associated code (e.g., study app, algorithms) produced by 4YouandMe will be made available via GitHub. Please check the 4YouandMe website (www.4youandme.org) or contact the corresponding author (sarah@4youandme.org) for an update on when study code will be available and how to access.

## References

[1] Moran PS (2020). Economic burden of maternal morbidity - a systematic review of cost-of-illness studies. PLoS ONE.

[2] Gon G (2018). The frequency of maternal morbidity: A systematic review of systematic reviews. Int. J. Gynaecol. Obstet..

[3] Global Burden of Disease, Injury Incidence and Prevalence Collaborators. (2018). Global, regional, and national incidence, prevalence, and years lived with disability for 354 diseases and injuries for 195 countries and territories, 1990-2017: a systematic analysis for the Global Burden of Disease Study 2017. Lancet.

[4] Tikkanen, R. G. M., FitzGerald M. & Zephyrin L. *Maternal Mortality and Maternity Care in the United States Compared to 10 Other Developed Countries*. *Issue briefs* (Commonwealth Fund, 2020).

[5] Magnus MC, Wilcox AJ, Morken NH, Weinberg CR, Haberg SE (2018). Role of maternal age and pregnancy history in risk of miscarriage: prospective register based study. BMJ.

[6] Wadhwa PD, Buss C, Entringer S, Swanson JM (2009). Developmental origins of health and disease: brief history of the approach and current focus on epigenetic mechanisms. Semin. Reprod. Med..

[7] Roberts JM, Bell M (2013). If we know so much about preeclampsia, why haven’t we cured the disease?. J. Reprod. Immunol..

[8] Latendresse G (2009). The interaction between chronic stress and pregnancy: preterm birth from a biobehavioral perspective. J. Midwifery Women’s Health.

[9] Wadhwa PD (2001). Stress, infection and preterm birth: a biobehavioural perspective. Paediatr. Perinat. Epidemiol..

[10] Li Y, Dalton VK, Lee SJ, Rosemberg MS, Seng JS (2020). Exploring the validity of allostatic load in pregnant women. Midwifery.

[11] Riggan KA, Gilbert A, Allyse MA (2021). Acknowledging and addressing allostatic load in pregnancy care. J. Racial Ethn. Health Disparities.

[12] Goodday S, Friend S (2019). Unlocking stress and forecasting its consequences with digital technology. npj Digital Med..

[13] Yang CC, Chao TC, Kuo TB, Yin CS, Chen HI (2000). Preeclamptic pregnancy is associated with increased sympathetic and decreased parasympathetic control of HR. Am. J. Physiol. Heart Circ. Physiol..

[14] Millman AL (2011). Oxygen saturation as a predictor of adverse maternal outcomes in women with preeclampsia. J. Obstet. Gynaecol. Can..

[15] Grym K (2019). Feasibility of smart wristbands for continuous monitoring during pregnancy and one month after birth. BMC Pregnancy Childbirth.

[16] Penton-Voak ISMM, Looi CY (2017). Biased facial-emotion perception in mental health disorders: a possible target for psychological intervention?. Curr. Direct Psychol. Sci..

[17] Cormack F (2019). Wearable technology for high-frequency cognitive and mood assessment in major depressive disorder: longitudinal observational study. JMIR Ment. Health.

[18] Simblett S (2018). Barriers to and facilitators of engagement with remote measurement technology for managing health: systematic review and content analysis of findings. J. Med. Internet Res..

[19] Pratap A (2020). Indicators of retention in remote digital health studies: a cross-study evaluation of 100,000 participants. NPJ Digit. Med..

[20] O’Connor S (2016). Understanding factors affecting patient and public engagement and recruitment to digital health interventions: a systematic review of qualitative studies. BMC Med. Inform. Decis. Mak..

[21] Ghassemi M., et al. Unfolding physiological state: mortality modelling in intensive care units. *KDD* 75–84 (2014). 10.1145/2623330.2623742 PMC4185189 25289175

[22] Tonekaboni S. J. S., Campbell K., Duvenaud D. K., Goldenberg A. What went wrong and when? Instance-wise feature importance for time-series black-box models. *Advances in Neural Information Processing Systems*, 33, (2020).

[23] AlHanai T., Ghassemi M. Predicting latent narrative mood using audio and physiologic data. *AAAI Conference on Artificial Intelligence* (2017).

[24] Wang B (2014). Similarity network fusion for aggregating data types on a genomic scale. Nat. Methods.

[25] Liu, G. et al. Clinically accurate chest X-ray report generation *arXiv*10.48550/arXiv.1904.02633 (2019).

[26] Suresh, H. et al. Clinical intervention prediction and understanding using deep networks. *arXiv*10.48550/arXiv.1705.08498 (2017).

[27] Rampášek L, Hidru D, Smirnov P, Haibe-Kains B, Goldenberg A (2019). Dr. VAE: improving drug response prediction via modeling of drug perturbation effects. Bioinformatics.

[28] Harris PA (2009). Research electronic data capture (REDCap) – A metadata-driven methodology and workflow process for providing translational research informatics support. J. Biomed. Inf..

[29] Harris, P. A. et al. REDCap Consortium, The REDCap consortium: building an international community of software partners. *J. Biomed. Inform*. **95**, 103208 (2019). 10.1016/j.jbi.2019.103208 PMC7254481 31078660

[30] Rhodes A, Smith AD, Chadwick P, Croker H, Llewellyn CH (2020). Exclusively digital health interventions targeting diet, physical activity, and weight gain in pregnant women: systematic review and meta-analysis. JMIR Mhealth Uhealth.

[31] “The Adverse Childhood Experiences (ACE) Study”. cdc.gov. Atlanta, Georgia: Centers for Disease Control and Prevention, National Center for Injury Prevention and Control, Division of Violence Prevention (2014).

[32] Weathers, Huska, & Keane. PTSD CheckList – Civilian Version (PCL-C). National Center for PTSD - Behavioral Science Division. https://www.mirecc.va.gov/docs/visn6/3_ptsd_checklist_and_scoring.pdf (1994).

[33] Gosling SD, Rentfrow PJ, Swann WB (2003). A very brief measure of the big five personality domains. J. Res. Personal..

[34] Koren, G. et al. Motherisk-PUQE (pregnancy-unique quantification of emesis and (PUQE) scoring index to assess severity of nausea and vomiting of pregnancy. *Am J Obstet Gynecol* **198**, 71.e1–71.e7 (2008).

[35] Yu L (2011). Development of short forms from the PROMIS™ Sleep Disturbance and Sleep-Related Impairment item banks. Behav. Sleep. Med..

[36] National Association of Community Health Centers, Inc. *PRAPARE: Protocol for Responding to and Assessing Patient Assets, Risks, and Experiences*. https://www.nachc.org/. http://www.nachc.org/wp-content/uploads/2016/09/PRAPARE_Paper_Form_Sept_2016.pdf (2016).

[37] Kroenke K, Spitzer RL, Williams JB (2001). The PHQ-9: validity of a brief depression severity measure. J. Gen. Intern Med.

[38] Posner K (2011). The Columbia–Suicide Severity Rating Scale: initial validity and internal consistency findings from three multisite studies with adolescents and adults. Am. J. psychiatry.

[39] Spitzer RL, Kroenke K, Williams JBW, Löwe B (2006). A Brief Measure for Assessing Generalized Anxiety Disorder: The GAD-7. Arch. Intern Med.

[40] Hays RD, Bjorner J, Revicki RA, Spritzer KL, Cella D (2009). Development of physical and mental health summary scores from the Patient Reported Outcomes Measurement Information System (PROMIS) global items. Qual. Life Res..

[41] Cella D (2007). The patient-reported outcomes measurement information system (PROMIS): Progress of an NIH roadmap cooperative group during its first two years. Med. Care.

[42] Pascoe JM, Ialongo NS, Horn WF, Reinhart MA, Perradatto D (1988). The reliability and validity of the maternal social support index. Fam. Med.

[43] Daltroy LH, Liang MH, Fossel AH, Goldberg MJ (1998). The POSNA pediatric musculoskeletal functional health questionnaire: report on reliability, validity, and sensitivity to change. Pediatric Outcomes Instrument Development Group. Pediatric Orthopaedic Society of North America. J. Pediatr. Orthop..

[44] Shrestha SD, Pradhan R, Tran TD, Gualano RC, Fisher JR (2016). Reliability and validity of the Edinburgh Postnatal Depression Scale (EPDS) for detecting perinatal common mental disorders (PCMDs) among women in low-and lower-middle-income countries: a systematic review. BMC pregnancy childbirth.

