# Supplementary material for: Better Understanding of the Metamorphosis of Pregnancy (BUMP): protocol for a digital feasibility study in women from preconception to postpartum
Source: NPJ Digit Med. 2022 Mar 30;5:40. doi: 10.1038/s41746-022-00579-9 (PMC8967890; doi:10.1038/s41746-022-00579-9)
Supplement: Supplementary file 1 — Supplementary Information [file 41746_2022_579_MOESM1_ESM.pdf]

Supplementary Note 1 - Recruitment Material Sample

[Subject]

[First Name], *Contribute to conception and pregnancy research--join a virtual study.*

[Header]
Pave the way for healthier moms and babies with your participation.

[Body]
Hi [First Name],

Sema4 is committed to improving outcomes for mothers and babies before, during and after pregnancy. Your input is essential to that endeavor. That's why we're helping the non-profit organization *4YouandMe* conduct a remote study that aims to detect and track pregnancy and conception symptoms and experiences.

We're inviting you to participate in this study because you recently took a pregnancy-related test with Sema4 and consented to receive information about our clinical studies. If you are trying to conceive or are currently pregnant, your insight could help advance what we know about conception, pregnancy, and beyond.

If you enroll, you'll be provided with a smart watch and potentially other wearable devices. You'll track your symptoms and experiences in just a few minutes each day using a smartphone app. *4YouandMe* will also offer knowledge and support during the study through regular phone or video calls.

If you're eligible and opt to participate, you'll be compensated for your time and may be able to keep one of the study devices.

If you'd like to learn more about the study, please visit the study page at clinicaltrials.gov. If you're interested in participating in this study, please fill out a brief survey.

Thank you,
Sema4
4YouandMe

CTA: I'm interested
